# *Centella asiatica* increases hippocampal synaptic density and improves memory and executive function in aged mice

**DOI:** 10.1002/brb3.1024

**Published:** 2018-06-19

**Authors:** Nora E. Gray, Jonathan A. Zweig, Maya Caruso, Marjoen D. Martin, Jennifer Y. Zhu, Joseph F. Quinn, Amala Soumyanath

**Affiliations:** ^1^ Department of Neurology Oregon Health and Science University Portland Oregon; ^2^ Department of Cell, Developmental and Cancer Biology Oregon Health and Science University Portland Oregon; ^3^ Department of Behavioral Neuroscience Oregon Health and Science University Portland Oregon; ^4^ Department of Neurology and Parkinson's Disease Research Education and Clinical Care Center (PADRECC) VA Portland Healthcare System Portland Oregon

**Keywords:** aging, antioxidant, memory

## Abstract

**Introduction:**

*Centella asiatica* is a plant used for centuries to enhance memory. We have previously shown that a water extract of *Centella asiatica* (CAW) attenuates age‐related spatial memory deficits in mice and improves neuronal health. Yet the effect of CAW on other cognitive domains remains unexplored as does its mechanism of improving age‐related cognitive impairment. This study investigates the effects of CAW on a variety of cognitive tasks as well as on synaptic density and mitochondrial and antioxidant pathways.

**Methods:**

Twenty‐month‐old CB6F1 mice were treated with CAW (2 mg/ml) in their drinking water for 2 weeks prior to behavioral testing. Learning, memory, and executive function were assessed using the novel object recognition task (NORT), object location memory task (OLM), and odor discrimination reversal learning (ODRL) test. Tissue was collected for Golgi analysis of spine density as well as assessment of mitochondrial, antioxidant, and synaptic proteins.

**Results:**

CAW improved performance in all behavioral tests suggesting effects on hippocampal and cortical dependent memory as well as on prefrontal cortex mediated executive function. There was also an increase in synaptic density in the treated animals, which was accompanied by increased expression of the antioxidant response gene NRF2 as well as the mitochondrial marker porin.

**Conclusions:**

These data show that CAW can increase synaptic density as well as antioxidant and mitochondrial proteins and improve multiple facets of age‐related cognitive impairment. Because mitochondrial dysfunction and oxidative stress also accompany cognitive impairment in many pathological conditions this suggests a broad therapeutic utility of CAW.

## 1 INTRODUCTION

As the aging population in the United States continues to grow, so does the need for treating age‐related declines in health and cognitive function. The majority of elderly individuals experience some form of memory loss that affects their activities of daily life including episodic and source memory deficits (Cansino, 2009; Johnson et al., 2016; Park et al., 2002), decreased sensitivity to novelty (Fandakova, Lindenberger, & Shing, 2014) and impairments in executive function tasks, including attention, planning, inhibitory control, and cognitive flexibility (Buckner, 2004; Methqal et al., 2017; Park et al., 2002). Similar deficits are observed in aged rodents (Burke, Wallace, Nematollahi, Uprety, & Barnes, 2010; Dalley, Cardinal, & Robbins, 2004; Gilbert et al., 2009; McQuail & Nicolle, 2015; Shimizu, Oki, Mitani, Tsuchiya, & Nabeshima, 2016; Young, Powell, Geyer, Jeste, & Risbrough, 2010).

The healthy aging brain displays mitochondrial abnormalities, like decreased mitochondrial content and reduced electron transport chain (ETC.) activity along with increased levels of reactive oxygen species (ROS) and markers of oxidative damage (Haider et al., 2014; Liu, Atamna, Kuratsune, & Ames, 2002). Studies have also demonstrated a relationship between mitochondrial function, antioxidant capacity, and memory (Forster et al., 1996; Masiero & Sandri, 2010; Olsen, Johnson, Zuloaga, Limoli, & Raber, 2013; Perrig, Perrig, & Stähelin, 1997) sparking an interest in identifying agents that target mitochondria and antioxidant pathways for the improvement of cognitive function.

The plant *Centella asiatica* (L) Urban, (Apiaceae), known in the United States as Gotu Kola, is used in traditional Chinese and Ayurvedic medicine to improve cognitive function (Kapoor, 1990; Shinomol, Muralidhara, & Bharath, 2011). Extracts of the plant have widely reported neuronal antioxidant and mitoprotective effects in vitro and in vivo (Gray, Harris, Quinn, & Soumyanath, 2016; Gray, Zweig, Matthews, et al., 2017; Gray, Zweig, Murchison, et al., 2017; Gupta & Flora, 2006; Haleagrahara & Ponnusamy, 2010; Ponnusamy, Mohan, & Nagaraja, 2008; Prakash, 2013; Shinomol & Muralidhara, 2008).

The cognitive enhancing effects of the plant have been supported by a handful of small clinical trials in healthy middle aged and older adults (Dev, Hambali, & Samah, 2009; Wattanathorn et al., 2008). A number of preclinical studies have also demonstrated similar cognitive enhancing effects of *Centella asiatica* in multiple rodent models of pathological cognitive impairment (Gupta & Srivastava, 2003; Kumar & Gupta, 2002; Soumyanath et al., 2012; Veerendra Kumar & Gupta, 2003). Our laboratory has previously shown that a water extract of *Centella asiatica* (CAW) added to the drinking water can attenuate spatial memory impairments in healthy aged mice (Gray et al., 2016).

Yet the effects of CAW on cognitive domains beyond spatial memory remains relatively unexplored as does its mechanism of improving age‐related cognitive impairment. Here we explore the effects of CAW on multiple cognitive domains beyond spatial memory, including recognition memory and executive function in healthy aged mice. We also examine the effects of the extract on synaptic density as well as mitochondrial and antioxidant protein expression in the brains of treated animals.

## 2 MATERIALS AND METHODS

### 2.1 Aqueous extract of Centella asiatica

Dried leaves of *Centella asiatica* was purchased (Oregon's Wild Harvest, GOT‐03193c‐OHQ01) and its identity was confirmed by comparing its thin layer chromatographic profile with that reported in the literature (Günther & Wagner, 1996) and the *Centella asiatica* samples used in our previous studies (Gray et al., 2014; Gray et al., 2016; Soumyanath et al., 2012). CAW was prepared by refluxing *Centella asiatica* (160 g) with water (2,000 ml) for 2 hr, filtering the solution and freeze drying to yield a powder (~16–21 g).

### 2.2 Animals

**Figure 1:**
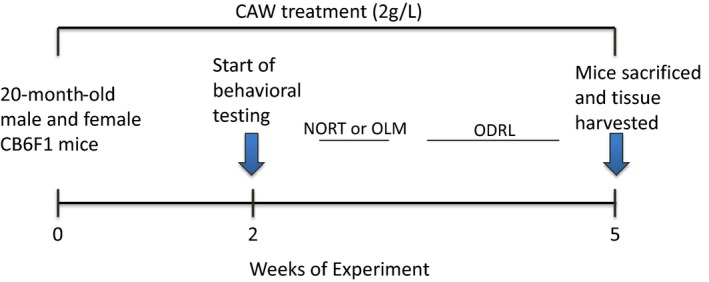
Timeline of CAW treatment and behavioral assessment. Mice were treated with CAW 2 weeks prior to the beginning of behavioral testing and treatment continued throughout the experiment. After testing, animals were sacrificed and tissue was harvested. CAW treatment lasted a total of 5 weeks

Twenty‐month‐old male and female CB6F1 mice were obtained from the NIH National Institute on Aging (NIA) aged rodent colony. Mice were maintained in a climate‐controlled environment with a 12‐hr light/12‐hr dark cycle, and fed AIN‐93M Purified Rodent Diet (Dyets Inc., Bethlehem, PA). Diet and water were supplied ad libitum. Mice were exposed to CAW in their drinking water (2 g/L) for 2 weeks prior to the beginning of behavioral testing. Control animals were given normal, unsupplemented drinking water. Thirty‐six mice (18 male, 18 female) were randomly assigned to treatment groups. Water consumption was monitored throughout the experiment to ensure the addition of CAW did not affect overall water intake. Following 3 weeks of behavioral testing, animals were sacrificed and tissue harvested as outlined in the timeline below (Figure 1). All mice completed behavioral testing and thus none were excluded from analysis. Based on pilot experiments we expected to see changes of 20%–25% in the behavioral tests after CAW treatment (standard deviation ~5%). Based on these estimates, we calculated 5–8 animals per condition to obtain adequate power with the Odor Discrimination Reversal Learning (ODRL) task requiring the most animals due to more subtle changes observed in our pilot experiments. Because of somewhat inconsistent availability of the CB6F1 mice from the NIA colony animals were run in two cohorts of 4–5 animals of each gender per treatment condition in each cohort. Each cohort was tested in Odor Discrimination reversal learning and either Novel Object Recognition or Object Location Memory. All animals were evaluated for protein expression changes and a subset of animals (3 of each gender per treatment condition) was also assessed by Golgi analysis. All procedures were conducted in accordance with the NIH Guidelines for the Care and Use of Laboratory Animals and were approved by the Institutional Animal Care and Use Committee of the Portland VA Medical Center.

### 2.3 Behavioral testing

Novel Object Recognition Task (NORT): This test takes advantage of the exploratory nature of mice and evaluates recognition memory. The experimental apparatus consisted of a white rectangular open field (39 cm x 39 cm x 39 cm). Habituation took place by exposing the animal to the experimental apparatus for 5 min in the absence of objects one time, on the day before training. During the training phase, mice were placed in the experimental apparatus in the presence of two identical objects and allowed to explore for three 10 min sessions. After 2 hr and 24 hr, mice were placed again in the apparatus, where this time one of the objects was replaced by a novel one (distinct at each time point). Mice were allowed to explore for 5 min. Preference for the novel object was expressed as the percent time spent exploring the novel object relative to the total time spent exploring both objects. The objects were a glass cylindrical votive, a metal cylindrical jar, a plastic rectangular box, and a plastic trapezoidal prism, all with approximately the same height. The identity of the objects—which one was novel or familiar—was balanced between groups. No preference was observed in this study for any object over the others.

Object Location Memory task (OLM): The OLM also capitalizes on the exploratory nature of mice and evaluates location memory. The experimental apparatus, habituation, and training for this task were identical as described above for the NORT. For the testing phases after 2 hr or 24 hr, mice were placed back in the apparatus but one of the objects was displaced to a novel spatial location (a third location was used at 24 hr). Mice were again allowed to explore the environment for 5 min Time spent exploring the displaced and nondisplaced objects was measured. Exploration was analyzed during both the training and testing phases.

In both the NORT and the OLM, objects were cleaned between trials with chlorhexidine (Nolvasan) to eliminate odor cues. All testing and training sessions were videotaped and analyzed by an experimenter blind to the treatment of the animals. It was considered exploration of the objects when mice were facing and sniffing the objects within very close proximity and/or touching. Mouse behavior was recorded with a video camera positioned over the behavioral apparatus and the collected videos were analyzed with the ANY‐MAZE software (Stoelting Co., Wood Dale, IL, USA).

Odor Discrimination Reversal Learning test (ODRL): This task, also called attention set‐shifting task, evaluates executive function. The test is comprised of four phases: shaping, learning, acquisition, and shift. Mice can be readily trained to dig in small bowls to retrieve food rewards (Bissonette et al., 2008; Young, Sharkey, & Finlayson, 2009; Young et al., 2007). Plastic cups (4.5 × 3 cm) were used for digging bowls and filled with a digging material of home cage bedding, dried black beans, or alder wood chips. The digging material was scented with lavender, mint or vanilla all commercially available (Fred Myers brand). The food reward was a part of a Froot Loop for each correct trial. The test apparatus was a gray rigid PVC enclosure (12″ × 8″7″) with a removable divider in the center. The digging bowls were placed on one side of the divider and the mouse on the other. At the beginning of each trial, the divider was removed.

In the shaping phase, mice were introduced to the testing chamber and trained to dig for a food reward in lavender scented bedding material. Mice were presented with a single bowl containing the food reward that was progressively filled with bedding in five stages, 0%, 25%, 50%, 75%, and 100% filled. The mouse advanced to the subsequent training step when it had successfully retrieved the food reward 5 times in a row.

The acquisition phase began after mice had completed the shaping phase. In this phase, mice were presented with two cups one containing dried beans and the other wood chips. In every trial, one digging material had the vanilla odor and the other the mint odor and the odor and material pairings were randomly alternated between trials but balanced over the acquisition phase so that each mouse was exposed to roughly equal combinations of each odor and digging material. Whether the baited cup was presented on the right or left side of the apparatus was also balanced throughout testing. In the acquisition phase, the mint‐scented bowl was always baited regardless of digging material. Example trials are found in Table 1. Each trial was initiated by raising the divider and allowing access to both bowls. Mice were required to make eight correct digs in any bout of 10 in order to reach criteria. Trials to criteria and latency to retrieve the reward were recorded.

**Table 1:** Example of test pairings for Odor Discrimination Reversal Learning (ODRL) test

	Right position	Left position
Acquisition phase	D1 + O1	*D2 + O2*
	*D1 + O2*	D2 + O1
	D2 + O1	*D1 + O2*
	*D2 + O2*	D1 + O1
Shift phase	*D1 + O1*	D2 + O2
	*D1 + O2*	D2 + O1
	D2 + O1	*D1 + O2*
	D2 + O2	*D1 + O1*

*Notes*

Representative combinations of odor and digging material pairings during each phase of the ODRL.

D1: dried bean; D2: wood chips; O1: vanilla; O2 = mint.

Italicized indicates correct trial.

After a mouse reached criteria in the acquisition phase, they immediately proceeded to the shift phase. As in the previous phase in the shift phase were presented with two cups one containing dried beans and the other wood chips. In every trial, one digging material had the vanilla odor and the other the mint odor and again the odor + digging material pairings were balanced throughout the trial as was right/left location of the baited cup. In the shift phase, however, the cup with the dried beans was always baited regardless of odor. Again, criteria were defined as eight correct trials in any bout of 10 and trials to criteria as well as latency to retrieve the reward was recorded. Mice were food restricted the night before each phase of the ODRL in order to motivate the animals.

### 2.4 Golgi

The FD Rapid GolgiStain™ Kit (FD Neurotechnologies) was used as per the manufacturer's instructions. In brief, one hemisphere of the brain was fixed for 9 days, sectioned coronally into 200um slices on a vibratome and mounted on gel‐coated slides. After drying, slides were stained and coverslipped using Permount (Fisher Scientific). Images were acquired using an Axio Imager M2 with an Apotome™ attachment and two cameras; an Axiocam 512 color and an AxioCam 506 mono. The system is driven by Zen 2™ software. Images taken with an EC Plan‐Neofluar40×/1.3 DIC WD = 0.21 objective in 5 phases and processed with a weak‐medium filter. Large area images were acquired with the Tiles module in a 2 × 2 array and stitched in the software. This system is part of the Advanced Light Microscopy Core at the Jungers Center, OHSU Portland, Oregon. Dendritic spines on pyramidal neurons from the CA1 region of the hippocampus were counted using Image J software (http://rsbweb.nih.gov/ij). Between three and six neurons were analyzed per animal and at least 50uM of clearly visible dendritic length was quantified per cell as previously described (Del Valle et al., 2012). To avoid bias, images were acquired and analyzed by two separate individuals both of whom were blinded to treatment conditions of the samples were analyzed per animal and at least 50uM of clearly visible dendritic length was quantified per image.

### 2.5 Western blot

Tissue (whole hippocampus) was homogenized and boiled in Laemmli buffer. Samples were separated electrophoretically on an SDS gel, transferred onto nitrocellulose membranes and immunoblotted using antibodies for NRF2 (Nuclear factor (erythroid‐derived 2)‐like 2, also called NFE2L2, Abcam Cat# ab62352, http://scicrunch.org/resolver/AB_944418), phosphorylated NRF2 (S40) (Abcam Cat# ab76026, http://scicrunch.org/resolver/AB_1524049), synaptophysin (Abcam Cat# ab14692, http://scicrunch.org/resolver/AB_301417), porin (also known as VDAC1, Abcam Cat# ab15895, http://scicrunch.org/resolver/AB_2214787), and GAPDH (Thermo Fisher Scientific Cat# MA5‐15738, http://scicrunch.org/resolver/AB_10977387). The optical density of the bands was quantified using Image J software and normalized to GAPDH.

### 2.6 Graphs and statistics

All bar graphs have error bars reflecting standard error of the mean. Statistical significance was determined using one‐ or two‐way analysis of variance or with appropriate *t* tests. Bonferroni post hoc tests were also conducted. Significance was defined as *p* ≤ 0.05. Analyses were performed using Excel or GraphPad Prism 6.

## 3 RESULTS

### 3.1 CAW improves location memory in aged mice

**Figure 2:**
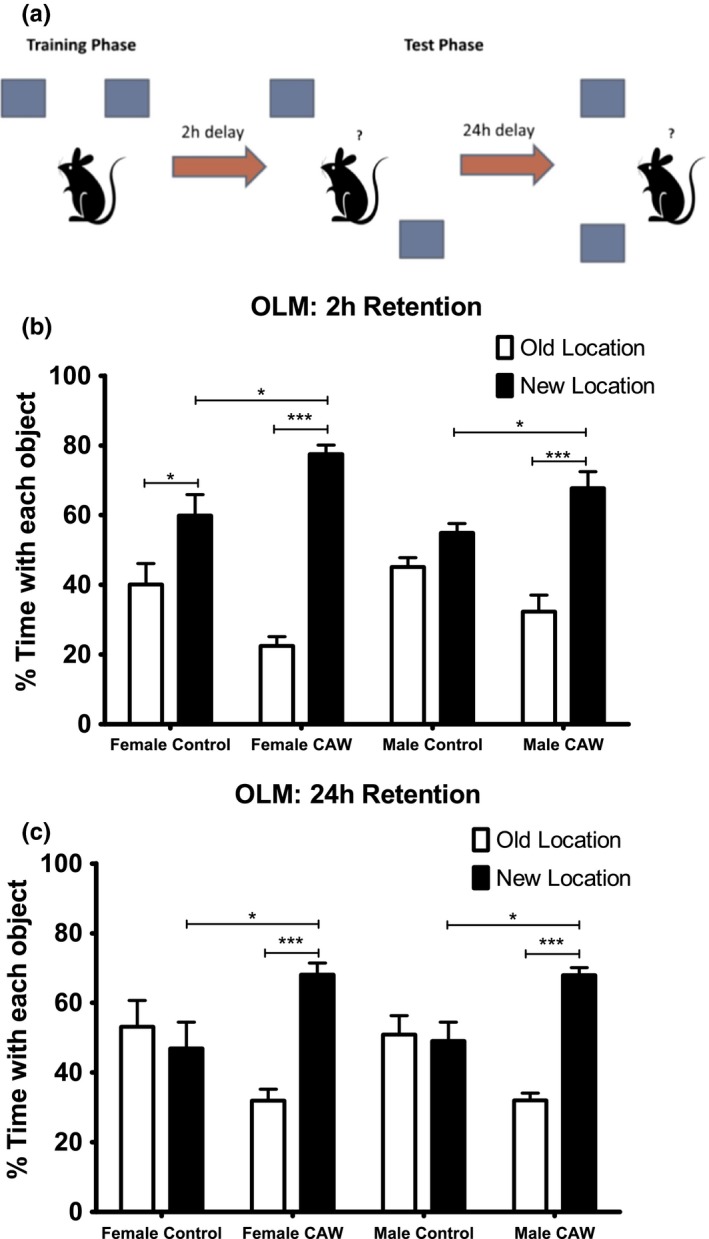
CAW treatment improves object location memory retention in aged mice. (a) Schematic of the Object Location Memory task (OLM) set up. (b) At the 2 hr retention time point CAW‐treated male and female mice had an enhanced preference for the novel location (*F* = 14.59 and 26.82 respectively). (c) A similar enhancement was also apparent in both genders at the 24 hr time point (*F* = 12.86 males, *F* = 5.801 females). *n* = 5 in each group, *p < 0.05, ***p < 0.001

We previously demonstrated the CAW improves spatial memory in aged C57BL6 mice (Gray et al., 2016). To validate this finding in the CB6F1 mouse line we used the OLM test. Aged CB6F1 mice (20 months) were treated with CAW in their drinking water (2 g/L) for 2 weeks prior to behavioral testing, and exposure to CAW continued throughout testing (Figure 1). CAW treatment improved performance in the OLM in both genders (Figure 2a). At 2 hr post‐training, a slight preference for the novel location was evident in the control mice but this preference was dramatically increased in the CAW‐treated male and female animals to males spending 68% and females 78% of their 5‐min test time with the new location (Figure 2b). Similar results were seen 24 hr post‐training. At this time point while control animals spent equal time exploring both locations, CAW‐treated mice spent far more time exploring the object in the new location, 68% of the time for both genders (Figure 2c).

### 3.2 CAW improves recognition memory in aged mice

**Figure 3:**
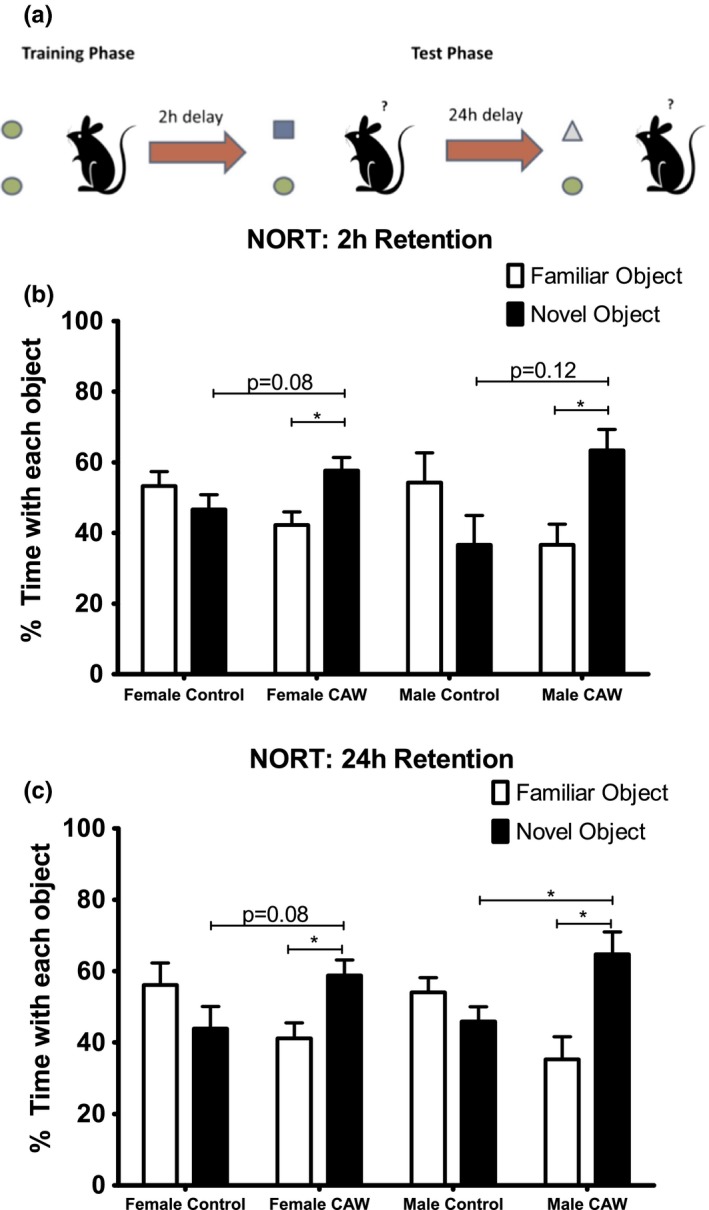
CAW treatment improves recognition memory retention in aged mice. (a) Schematic of the Novel Object Recognition Task (NORT) set up. (b) Control treated animals had no preference for the novel object at the 2 hr time point while there was a significant preference for the novel object in CAW‐treated male and female mice (*F* = 2.487 and 3.098, respectively). (c) The novel object preference was also observable after 24 hr in the CAW‐treated mice (*F* = 5.497 males, *F* = 2.703 females). *n* = 5 in each group, *p < 0.05

CAW treatment also improved performance in the NORT in both male and female mice (Figure 3a). While control animals did not display a preference for the novel object at either 2 hr or 24 hr post‐training, CAW‐treated animals spent significantly more time exploring the novel object than the familiar object at both time points, with males and females spending 63% and 58% of the 5 min test time, respectively, at 2 hr and 65% and 59% of their time, respectively, after 24 hr (Figure 3b,c).

### 3.3 CAW improves learning and executive function in aged mice

**Figure 4:**
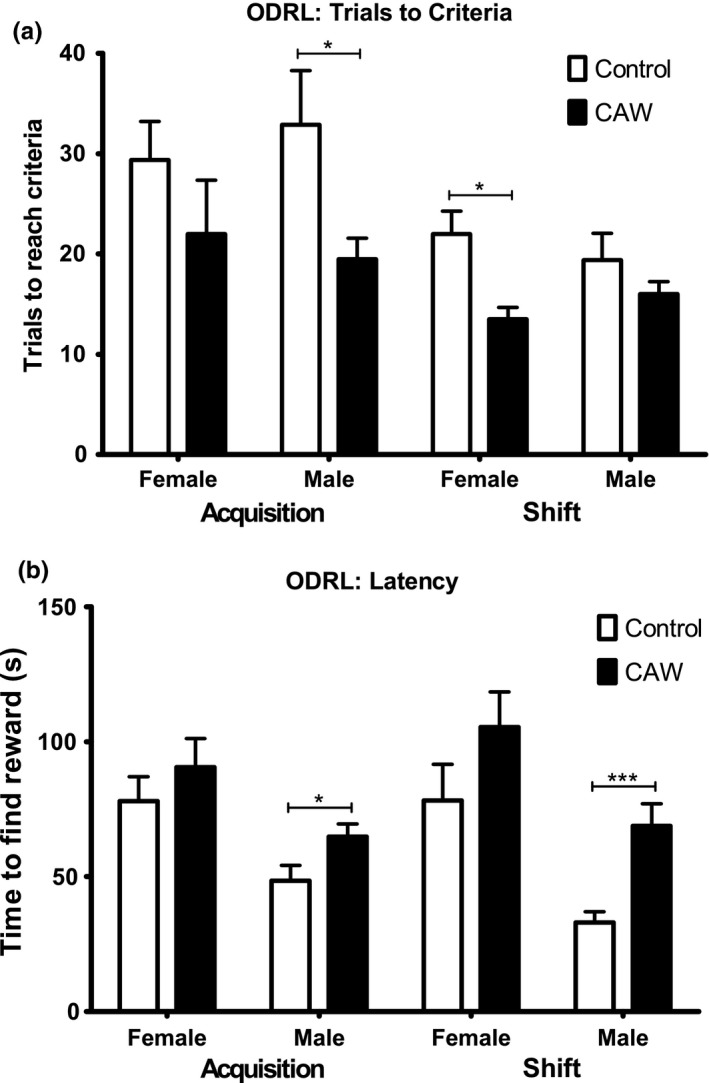
CAW treatment improves executive function in aged mice. (a) In the acquisition phase of the ODRL CAW reduced the number of trials to reach criteria in male aged mice. In the shift phase of the test, there was also a reduction in the number of trials to reach criteria in the CAW‐treated animals although this only reached significance in the female mice (*F* = 4.618 males, *F* = 3.369 females). (b) The time to find the reward was significantly increased by CAW treatment in the male animals in both the acquisition and shift phases (*F* = 3.750). *n* = 8 in each group, *p < 0.05, ***p < 0.001

The ODRL, also called attention set‐shifting task, evaluates executive function. Executive function includes behaviors like attentional selection, behavioral inhibition, cognitive flexibility, task switching, planning, and decision‐making (Buckner, 2004). The two test components of the OD RL are the acquisition and shift phases. While the acquisition phase assesses learning, the shift phase probes executive function, specifically cognitive flexibility. In the acquisition phase of the ODRL male CAW‐treated mice took significantly fewer trials than controls to reach criteria (Figure 4a). Female CAW‐treated mice showed a similar trend toward improved performance in the acquisition phase but it did not achieve significance. In contrast, in the shift phase, CAW‐treated female mice needed significantly fewer trials to reach criteria than their control counterparts. In the male mice, CAW treatment reduced the number of trials necessary as well but not significantly (Figure 4a). Interestingly in male mice, CAW treatment also significantly increased the latency retrieve the reward in both the acquisition and shift phases. Female CAW‐treated mice showed a similar trend toward increased latency but it was not significant in either test phase (Figure 4b).

### 3.4 CAW increases synaptic density in the hippocampus of aged mice

**Figure 5:**
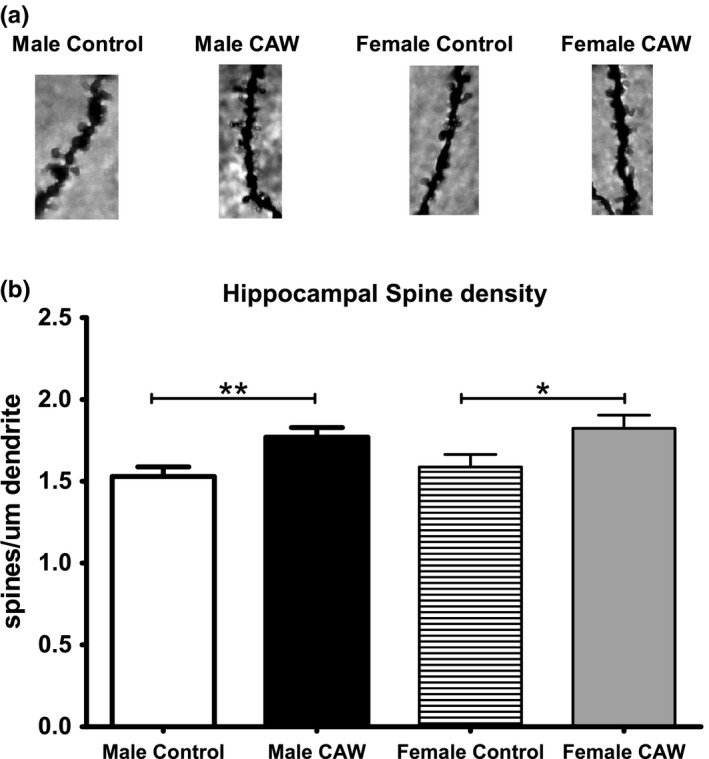
CAW increases spine density in the hippocampus of aged mice. (a) Example images of dendritic spines on hippocampal neurons from aged animals. (b) Quantification of spine density. Three animals were evaluated per treatment condition with 3–6 images quantified per animal. *p < 0.05, **p < 0.01

**Figure 6:**
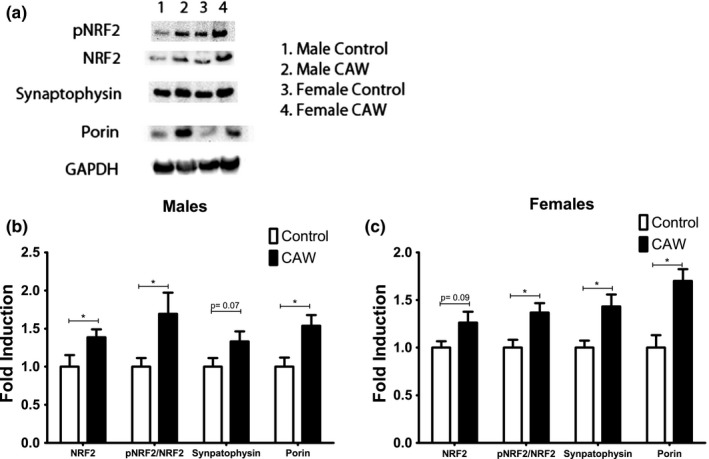
CAW increases antioxidant, mitochondrial, and synaptic proteins in the hippocampus of aged mice. (a) Representative western blot from aged animals. (b) Quantification of multiple blots. CAW increased the expression of NRF2 protein as well as the ratio of phosphorylated NRF2 to total NRF2 in the hippocampus of aged male mice. CAW treatment also significantly increased expression of the mitochondrial protein porin (*F* = 41.28). (c) Quantification of multiple blots. CAW increased the ratio of phosphorylated NRF2 to total NRF2 in the brains of aged female mice. The expression of porin and synaptophysin were similarly increased in these animals (*F* = 31.03). *n* = 8–9 in each group, *p < 0.05

CAW treatment resulted in a significant increase in dendritic spine density in the CA1 region of hippocampus in male mice. There was a comparable increase in hippocampal spine density in CAW‐treated female mice (Figure 5a,b). CAW also increased the hippocampal expression of the presynaptic protein synaptophysin in female mice when normalized to GAPDH. A similar trend was observed in CAW‐treated male mice although it did not reach significance (Figure 6a–c).

### 3.5 CAW increases hippocampal expression of antioxidant and mitochondrial proteins in aged mice

CAW increased the expression of the antioxidant regulatory protein NRF2 in the hippocampus of male and female mice (Figure 6a–c). The ratio of phosphorylated NRF2 to total NRF2 was also increased both genders. The mitochondrial protein porin (also called VDAC1) was robustly increased in the brains of the CAW‐treated animals.

## 4 DISCUSSION

The beneficial effects of CAW on neuronal health and cognitive function have been well‐documented both in vitro and in vitro (Gupta & Flora, 2006; Gupta & Srivastava, 2003; Kumar & Gupta, 2002; Shinomol & Muralidhara, 2008; Soumyanath et al., 2005; Veerendra Kumar & Gupta, 2003). Our laboratory has previously reported that CAW improves performance in the Morris Water Maze (MWM) in mice exposed to Aβ as well as healthy older mice (Gray et al., 2016; Soumyanath et al., 2012). In this study, we further explore the effects of CAW on age‐related cognitive impairment using a battery of behavioral tests assessing learning, memory, and executive function.

We found that treatment with CAW for 2 weeks prior to the beginning of, and continuing throughout behavioral testing (Figure 1), improved the performance of both male and female 20‐month‐old CB6F1 mice in the OLM test. The OLM is a hippocampal‐dependent test of location memory (Assini, Duzzioni, & Takahashi, 2009; Cipolotti, 2006) which is known to be impaired with aging in both humans and rodents (Arias‐Cavieres, Adasme, Sánchez, Muñoz, & Hidalgo, 2017; Sapkota, van der Linde, Lamichhane, Upadhyaya, & Pardhan, 2017; Wimmer, Hernandez, Blackwell, & Abel, 2012). The improvement in the OLM seen in this study is consistent with previous studies showing that asiatic acid, a major triterpene component of *Centella asiatica* (Siddiqui, Aslam, Ali, Khan, & Begum, 2007), improves performance in the same task in healthy as well as cognitively impaired rodents (Chaisawang et al., 2017; Sirichoat et al., 2015; Umka Welbat et al., 2016). It is also in line with reports that OLM performance by aged mice is improved by treatment with polyphenols (Carey, Gomes, & Shukitt‐Hale, 2014; Matsui et al., 2009), a class of compounds which both *Centella asiatica* in general (Siddiqui et al., 2007; Subban, Veerakumar, Manimaran, Hashim, & Balachandran, 2007) and CAW specifically are rich in (Gray, Zweig, Matthews, et al., 2017; Gray, Zweig, Murchison, et al., 2017; Soumyanath et al., 2012). It is likewise consistent with our laboratory's previous finding that CAW improves MWM performance, another hippocampal‐dependent task, in healthy aged mice (Gray et al., 2016). Interestingly in that task, there was a more pronounced effect of CAW in the male animals while in the OLM no gender differences were observed.

We also observed improvements in the NORT performance following CAW treatment in aged CB6F1 mice. This suggests that the beneficial effects of CAW may not be restricted to the hippocampus as both the hippocampus and cortex are known to play an important role in object recognition memory (Aggleton, Albasser, Aggleton, Poirier, & Pearce, 2010; Buckmaster, Eichenbaum, Amaral, Suzuki, & Rapp, 2004; Clark, Zola, & Squire, 2000; Hammond, Tull, & Stackman, 2004). Age‐related impairments in object recognition are seen in both rodents and humans (Diaz et al., 2017; Kaviani et al., 2017; Li et al., 2015; Merriman, Ondřej, Roudaia, O'Sullivan, & Newell, 2016; Singh & Thakur, 2014). While to our knowledge this is the first report of *Centella asiatica* affecting recognition memory in aged rodents, it has been demonstrated that performance in the NORT is improved following administration of other polyphenol‐containing plant extracts (Carey et al., 2014; Matias et al., 2017; Nam et al., 2013; Yu et al., 2013).

This study is also the first report, to our knowledge, of effects of *Centella asiatica* on executive function. Executive function includes elements like impulse control, attention, planning, cognitive flexibility, and problem solving. It is mediated by the prefrontal cortex and is very sensitive to age‐related decline (Buckner, 2004; Raz & Rodrigue, 2006). The Wisconsin Card Sorting Test (WCST) is one of a number of widely used tests to assess executive function in humans. In this task subjects are required to adapt behavioral responses to choose the “correct” stimulus array based on sudden rule changes across multiple modalities (Eling, Derckx, & Maes, 2008). Performance in this task declines with age (Ashendorf & McCaffrey, 2008). The ODRL, also called attention set‐shifting task, is a parallel test that has been developed for rats and, more recently, mice (Birrell & Brown, 2000; Garner, Thogerson, Würbel, Murray, & Mench, 2006). Like the WCST the ODRL requires paying attention to relevant stimuli while ignoring irrelevant stimuli and subsequently shifting the attention, either within dimensions or between dimensions of the test stimuli (Birrell & Brown, 2000). Also like the WCST performance in the ODRL declines with age (Barense, Fox, & Baxter, 2002; Beas, Setlow, & Bizon, 2013; Young et al., 2010).

We found that CAW treatment improved performance in both the acquisition and shift phases of this test. The acquisition phase of the ODRL assesses classical learning and the improvement observed following CAW treatment is in line with our previous report of enhanced performance of CAW‐treated aged mice in the hidden platform phase of the MWM. The shift phase of ODRL is the metric of cognitive flexibility. Here we also observed an improvement following CAW treatment.

Interestingly while the number of trials to reach criteria in both ODRL phases decreased with CAW treatment, the latency to find the reward appeared to increase in treated animals, especially the treated males. This combination of effects could suggest decreased impulsivity in the CAW‐treated animals or an improved accuracy trade‐off strategy with a shift toward more goal‐directed action instead of habitual action. The selection of goal‐directed actions is governed by associations between the value of the consequences and is sensitive to changes in the causal relationship between the action and those consequences whereas habituation actions are controlled through stimulus‐response associations without the association with the value of the outcome (Griffiths, Morris, & Balleine, 2014). Imbalances between goal‐directed and habitual action are observed in many neurological disorders including Parkinson's disease, Tourette Syndrome and obsessive‐compulsive disorder (Gillan & Robbins, 2014; Pappas, Leventhal, Albin, & Dauer, 2014; Redgrave et al., 2010) so these results may indicate a broad therapeutic benefit of CAW beyond age‐related cognitive impairment. It would be interesting in future studies to see if similar effects are seen in young animals.

In this study, we also observed increased synaptic density in the CAW‐treated animals. We have previously demonstrated that CAW can increase spine density in primary hippocampal neurons in culture (Gray, Zweig, Matthews, et al., 2017; Gray, Zweig, Murchison, et al., 2017) but here we show oral administration of the extract exerts the same effects in vivo. In addition, the CAW‐induced an increase in the expression of synaptophysin is consistent with our previous report of increased gene expression of synaptophysin and postsynaptic density protein 95 the brains of aged CAW‐treated mice (Gray et al., 2016). As increased synaptic density is known to correlate with improved cognitive function (Terry et al., 1991) this is likely the physiological underpinning of the improvement in hippocampal‐dependent tests seen in this study. The fact that we also saw improvements in executive function suggests that these structural changes are likely occurring in other brain regions as well, specifically the prefrontal cortex. In fact, the changes in synaptic gene expression that we reported previously (Gray et al., 2016) were found to occur in multiple brain regions further supporting this idea.

The observed changes in NRF2 and porin are in accordance with our previous gene expression data as well (Gray et al., 2016). These increases suggest an activation of the antioxidant pathway and an increase in mitochondrial content respectively. It remains to be seen what contribution each of these play in the memory enhancing properties of CAW, but in mice cognitive decline is associated with dysfunctional mitochondria (Masiero & Sandri, 2010) and increased oxidative damage (Forster et al., 1996). Moreover, both over‐expressing antioxidant enzymes and increasing mitochondrial content has been shown to improve memory in rodents (Cao et al., 2010; Chen, Na, & Ran, 2014; Olsen et al., 2013). Experiments are underway in the laboratory to evaluate the effects of the extract on NRF2KO mice to determine if activation of the NRF2 pathway is required for the cognitive enhancing effects of CAW.

Our findings here further demonstrate the cognitive enhancing effects of CAW. Relatively short treatment with extract improved several different domains of cognitive performance in aged animals and enhanced synaptic density as well as mitochondrial and antioxidant response pathways in vivo. While the exact relationship between these effects remains to be elucidated, the fact that synaptic dysfunction and cognitive impairment accompany oxidative stress and mitochondrial dysfunction in many pathological conditions (Emerit & Bricaire, 2004; Lin & Beal, 2006) suggests the potential utility of CAW is far broader than for aging alone.

## CONFLICT OF INTEREST

None declared.
